# Time Course of Low-Frequency Oscillatory Behavior in Human Ventricular Repolarization Following Enhanced Sympathetic Activity and Relation to Arrhythmogenesis

**DOI:** 10.3389/fphys.2019.01547

**Published:** 2020-01-14

**Authors:** David Adolfo Sampedro-Puente, Jesus Fernandez-Bes, Norbert Szentandrássy, Péter Nánási, Peter Taggart, Esther Pueyo

**Affiliations:** ^1^BSICOS Group, I3A, IIS Aragón, University of Zaragoza, Zaragoza, Spain; ^2^Department of Physiology, Faculty of Medicine, University of Debrecen, Debrecen, Hungary; ^3^Department of Dental Physiology and Pharmacology, Faculty of Dentistry, University of Debrecen, Debrecen, Hungary; ^4^Department of Cardiovascular Sciences, University College London, London, United Kingdom; ^5^Center for Biomedical Research in the Network in Bioengineering, Biomaterials and Nanomedicine (CIBER-BBN), Zaragoza, Spain

**Keywords:** low-frequency oscillations, beta-adrenergic stimulation, cardiac cell models, ventricular repolarization, sympathetic activity, arrhythmogenesis

## Abstract

**Background and Objectives:** Recent studies in humans and dogs have shown that ventricular repolarization exhibits a low-frequency (LF) oscillatory pattern following enhanced sympathetic activity, which has been related to arrhythmic risk. The appearance of LF oscillations in ventricular repolarization is, however, not immediate, but it may take up to some minutes. This study seeks to characterize the time course of the action potential (AP) duration (APD) oscillatory behavior in response to sympathetic provocations, unveil its underlying mechanisms and establish a potential link to arrhythmogenesis under disease conditions.

**Materials and Methods:** A representative set of human ventricular computational models coupling cellular electrophysiology, calcium dynamics, β-adrenergic signaling, and mechanics was built. Sympathetic provocation was modeled via phasic changes in β-adrenergic stimulation (β-AS) and mechanical stretch at Mayer wave frequencies within the 0.03–0.15 Hz band.

**Results:** Our results show that there are large inter-individual differences in the time lapse for the development of LF oscillations in APD following sympathetic provocation, with some cells requiring just a few seconds and other cells needing more than 3 min. Whereas, the oscillatory response to phasic mechanical stretch is almost immediate, the response to β-AS is much more prolonged, in line with experimentally reported evidences, thus being this component the one driving the slow development of APD oscillations following enhanced sympathetic activity. If β-adrenoceptors are priorly stimulated, the time for APD oscillations to become apparent is remarkably reduced, with the oscillation time lapse being an exponential function of the pre-stimulation level. The major mechanism underlying the delay in APD oscillations appearance is related to the slow *I*_*Ks*_ phosphorylation kinetics, with its relevance being modulated by the *I*_*Ks*_ conductance of each individual cell. Cells presenting short oscillation time lapses are commonly associated with large APD oscillation magnitudes, which facilitate the occurrence of pro-arrhythmic events under disease conditions involving calcium overload and reduced repolarization reserve.

**Conclusions:** The time course of LF oscillatory behavior of APD in response to increased sympathetic activity presents high inter-individual variability, which is associated with different expression and PKA phosphorylation kinetics of the *I*_*Ks*_ current. Short time lapses in the development of APD oscillations are associated with large oscillatory magnitudes and pro-arrhythmic risk under disease conditions.

## 1. Introduction

Ventricular repolarization has been shown to exhibit a low-frequency (LF) oscillatory pattern following enhanced sympathetic activity. In humans, this has been demonstrated by quantification of so-called periodic repolarization dynamics in the T-wave vector of the electrocardiogram (ECG) (Rizas et al., 2014, 2016) as well as by *in vivo* evaluation of LF components in activation recovery intervals (ARI) of ventricular electrograms (Hanson et al., 2014; Porter et al., 2018). In post-infarction patients, an increased magnitude of LF oscillations in ECG repolarization has been proved to be a significant predictor of total mortality and sudden cardiac death (Rizas et al., 2017). Most notably, a very recent study has shown that those periodic repolarization dynamics are able to predict the efficacy of implanting a cardioverter defibrillator in patients undergoing primary prophylactic treatment (Bauer et al., 2019). *In silico* studies have provided insight into the cellular mechanisms underlying this oscillatory pattern of ventricular repolarization, which have been explained by the synergistic effect of phasic β-adrenergic stimulation (β-AS) and mechanical stretch, both accompanying enhanced sympathetic nerve activity. In brief, differential phosphorylation kinetics of calcium (*I*_*Ca*_) and potassium (*I*_*K*_) currents upon phasic β-AS as well as changes in calcium cycling and the action of stretch-activated channels (SACs) in response to phasic mechanical stretch have been shown to generate LF oscillations in cellular action potential (AP) duration (APD) (Pueyo et al., 2016). Subsequent studies have additionally investigated inter-individual differences in LF oscillations of ventricular APD, concluding that calcium and potassium currents, *I*_*Ca*_ and *I*_*K*_ (specifically, the rapid delayed rectifier *I*_*Kr*_ and inward rectifier *I*_*K*1_), are major ionic modulators of such inter-individudal differences (Sampedro-Puente et al., 2019). Importantly, these identified ionic factors are key for the development of arrhythmic events following enhancement of APD oscillations' magnitude. A very recent investigation has experimentally confirmed in an arrhythmogenic *in vivo* dog model that ventricular remodeling associated with chronic atrioventricular block (CAVB) augments LF oscillations of APD (Sprenkeler et al., 2019). Most importantly, the oscillation magnitude has been reported to be larger in dogs susceptible to dofetilide-induced Torsades de Pointes arrhythmias as compared to non-inducible dogs (Sprenkeler et al., 2019).

For LF oscillations in the ventricular APD to become clearly manifested following increased sympathetic activity, computational research has shown that some tens of seconds or even a few minutes are required (Pueyo et al., 2016). This requisite on a relatively long exposure to enhanced sympathetic activity for repolarization oscillations to develop may explain why experimentally measured APD oscillations appear to come and go and do not remain as sustained oscillations for long recording periods (Hanson et al., 2014). Pueyo et al. (2016) and Sampedro-Puente et al. (2019) have shown that, upon a sympathetic rise, the cellular ventricular APD shows a global trend of shortening, or brief prolongation followed by more prominent shortening, which masks concurrent LF oscillations overlapping with the global APD trend. The individual and combined roles of β-AS and mechanical stretch in determining the time lapse for LF oscillations to become visibly manifested are yet to be explored. Experimental investigations in canine ventricular myocytes have shown that APD presents slow time-dependent changes following application of a constant dose of the β-adrenergic agonist isoproterenol (ISO) (Ruzsnavszky et al., 2014). The slow activation of *I*_*K*_ currents (in particular, slow *I*_*Ks*_ and rapid *I*_*Kr*_ delayed rectifier currents), as compared to the very fast activation of the *I*_*Ca*_ current, has been demonstrated to be behind such APD lag following sudden ISO exposure. The distinctively slow response of *I*_*Ks*_ to β-AS and its implications in terms of APD adaptation time have been also described in other species, like the rabbit (Liu et al., 2012). On the other hand, APD dynamicity in response to constant mechanical stretch or to the combination of constant β-AS and mechanical stretch has been less studied experimentally.

The present study investigates the cellular ventricular APD response to phasic, rather than constant, β-AS and mechanical stretch, in closer correspondence with the experimentally reported LF patterns of efferent sympathetic nerve activity (Pagani et al., 1997; Furlan et al., 2000). The global trend of APD response is in this case expected to be concurrent with periodic changes in APD occurring at the frequency of sympathetic activity. For this investigation, a population of computational cellular AP models representative of experimentally reported human ventricular electrophysiological characteristics is developed and coupled to models of β-AS and mechanics. By using the developed models, the amount of time required for LF fluctuations of APD to arise in response to phasic sympathetic activation is characterized and the ionic mechanisms underlying cell-to-cell differences in APD time lag are dissected. Experimental confirmation of the obtained results is obtained. A relationship between the quantified time lapse and the magnitude of APD oscillations is established, which serves to set links to pro-arrhythmic risk under disease conditions associated with Ca^2+^ overload and reduced repolarization reserve (RRR), both being commonly present in failing hearts.

## 2. Methods

### 2.1. Experimental Data

Ventricular myocytes were isolated from the left ventricular wall of adult beagle dogs as described in Ruzsnavszky et al. (2014). The isolation procedure followed a protocol approved by the local ethical committee according to the principles outlined in the 1964 Declaration of Helsinki and its later amendments. The cells used for this study were obtained from the subepicardial layer.

Transmembrane potentials were measured at 37°C by using 3 M KCl-filled sharp glass microelectrodes with tip resistance 20–40 MΩ (Ruzsnavszky et al., 2014). The electrodes were connected to the input of an Axoclamp-2B amplifier (Molecular Devices, Sunnyvale, CA, USA). Cardiomyocytes were paced at 1 s using 1-ms wide rectangular current pulses with 120% threshold amplitude until steady-state. ISO was applied at a concentration of 10 nM for 5 min. APs were sampled by periods of 30 s following ISO application, with a sampling frequency of 200 kHz using Digidata 1200 A/D card (Axon Instruments Inc., Foster City, CA, USA).

### 2.2. Electrophysiology Model

A population of human ventricular AP models representative of a wide range of experimentally observed electrophysiological characteristics was built based on the O'Hara-Virág-Varró-Rudy (ORd) epicardial model (O'Hara et al., 2011). The population was obtained by varying the ionic conductances of eight ionic currents in the ORd model, namely: *I*_*Ks*_, slow delayed rectifier potassium current; *I*_*Kr*_, rapid delayed rectifier potassium current; *I*_*to*_, transient outward potassium current; *I*_*CaL*_, L-type calcium current; *I*_*K*1_, inward rectifier potassium current; *I*_*Na*_, sodium current; *I*_*NaK*_, sodium-potassium pump current; and *I*_*NaCa*_, sodium-calcium exchanger current.

Initially, 500 models were generated by using the Latin Hypercube Sampling method to sample the conductances of the above described currents in the range ±100% (McKay et al., 1979; Pueyo et al., 2016). A set of calibration criteria based on experimentally available human ventricular measures of steady-state AP characteristics (Jost et al., 2008; Grandi et al., 2010; Guo et al., 2011; O'Hara et al., 2011; Britton et al., 2017) were imposed, as described in Table 1. AP characteristics used for model calibration included: APD_90|50_, which represents steady-state AP duration (APD) at 90|50% repolarization corresponding to 1 Hz pacing (expressed in ms); RMP, representing resting membrane potential (in mV); *V*_peak_, representing peak membrane potential measured in the AP upstroke (in mV); and ΔAPD_90_, representing the percentage of change in APD_90_ with respect to baseline following individual inhibitions of *I*_*Ks*_, *I*_*Kr*_, or *I*_*K*1_ currents (measured in ms). Of the initial 500 models, only 218 meeting all the calibration criteria were selected. Additionally, models showing pro-arrhythmic behavior at baseline and/or under sympathetic provocation were discarded, which led to a population of 188 models for the analysis of this study.

**Table 1:** Calibration criteria applied onto human ventricular cell models.

**AP characteristic**	**Min. acceptable value**	**Max. acceptable value**
Under baseline conditions (Guo et al., 2011; O'Hara et al., 2011; Britton et al., 2017)		
APD_90_ (ms)	178.1	442.7
APD_50_ (ms)	106.6	349.4
RMP (mV)	−94.4	−78.5
*V*_peak_ (mV)	7.3	−
Under 90% *I*_*Ks*_ block (O'Hara et al., 2011)		
ΔAPD_90_ (%)	−54.4	62
Under 70% *I*_*Kr*_ block (Grandi et al., 2010)		
ΔAPD_90_ (%)	34.25	91.94
Under 50% *I*_*K*1_ block (Jost et al., 2008)		
ΔAPD_90_ (%)	−5.26	14.86

### 2.3. PKA Phosphorylation Model

A modified version of the Xie et al. (2013) β-adrenergic signaling model was used as a basis to describe phosphorylation levels of cellular protein kinase A (PKA) substrates, as described in Pueyo et al. (2016) and Sampedro-Puente et al. (2019). The Xie et al. (2013) model represents an evolution from the Soltis and Saucerman (2010) signaling model in which *I*_*K*_*s*__ phosphorylation and dephosphorylation rate constants were updated to better match experimental observations reported in Liu et al. (2012). Also, as described in Xie et al. (2013), PKA-mediated phosphorylation of phospholemman (PLM) involved an increase in the Na^+^-K^+^-ATPase (NKA) affinity for the intracellular Na^+^ concentration. In the modified Xie et al. (2013) model of this study, ryanodine receptors (RyR) phosphorylation was defined by using the formulation described in Heijman et al. (2011).

For a specific set of simulations, *I*_*K*_*s*__ phosphorylation and dephosphorylation kinetics were defined as reported in Soltis and Saucerman (2010) to assess the effects of faster phosphorylation kinetics on the time lapse for APD oscillations development.

### 2.4. Mechanics Model

An updated version of the Niederer et al. (2006) model was employed to describe cell mechanics, with the values of some constants being adjusted to represent human cell characteristics as in Weise and Panfilov (2013) and Pueyo et al. (2016). *I*_*SAC*_, denoting the current through SACs, was accounted for as in Pueyo et al. (2016). Specifically, *I*_*SAC*_ was defined as the current through non-specific cationic SACs plus the current through K^+^-selective SACs.

### 2.5. Simulation of Enhanced Sympathetic Activity

Enhanced sympathetic activity was simulated by the combination of phasic β-AS and mechanical stretch effects. Phasic β-AS was simulated by a periodic stepwise profile of the β-adrenergic agonist ISO according to muscle sympathetic nerve activity patterns in humans (Pagani et al., 1997). The periodicity of the ISO profile corresponded to a frequency of 0.05 Hz, this being within the reported Mayer wave frequency range (0.03–0.15 Hz). The 20 s ISO period was composed of a 10 s time interval where the ISO concentration was set to 1 μM and a subsequent 10 s time interval where the ISO concentration was 0. Phasic changes in hemodynamic loading, a known accompaniment of enhanced sympathetic activity, were simulated by phasic mechanical stretch changes at the same 0.05 Hz frequency. Specifically, stretch ratio was varied during the 20 s period by following a sinusoidal waveform with maximal change being 10%, being such level of change in line with those of previous experimental and computational studies (Niederer and Smith, 2007; Iribe et al., 2014). Phasic β-AS and mechanical stretch effects were defined to be in-phase. Five hundred beats at baseline and 500 beats following enhanced sympathetic activity were simulated while pacing at 1 Hz frequency.

### 2.6. Simulation of Disease Conditions

For specific simulations, disease conditions were simulated by Reduced Repolarization Reserve (RRR) and Ca^2+^ overload. RRR was defined by concomitant inhibition of *I*_*Kr*_ and *I*_*Ks*_ currents by 30 and 80%, respectively. Ca^2+^ overload was defined by a 4-fold increment in the extracellular Ca^2+^ level.

### 2.7. Quantification of APD Time Lag in Response to Constant β-AS and/or Mechanical Stretch

APD was evaluated at 90% repolarization in both simulations and experiments. The simulated or experimentally measured APD time series following β-AS and/or mechanical stretch is denoted by *a*[*k*], where the discrete index *k* represents cycle number. Thus, *k* varies from 0 to *K*, with *K* being the number of cycles following β-AS and/or mechanical stretch.

The time lapse, τ_APD_, for APD to reach a new steady-state following application of β-AS and/or stretch was defined as the time taken by the APD time series to attain convergence, with convergence characterized by the derivative of the APD time series being below a predefined threshold. Specifically, the following steps were used to compute the APD time lapse:

1. **Smoothing**
  To remove short-term variability and make the estimation of the convergence time more robust, moving average smoothing was applied onto the APD time series *a*[*k*] to obtain a smooth version of it, $\hat{a}\left[k\right]$:
  (1) $$\begin{array}{l} \hat{a}\left[k\right]=\frac{1}{T}\overset{k+T}{\underset{k^{\prime }=k}{\sum }}a\left[k^{\prime }\right] \end{array}$$
  where *T* was set to the period in cycles of the sympathetic activity, *T* = 20 cycles.
2. **Numerical derivative**
  From $\hat{a}\left[k\right]$, the derivative *d*[*k*] was numerically estimated by computing the central difference for the interior data points of $\hat{a}\left[k\right]$ and single-side difference for the edges of $\hat{a}\left[k\right]$:
  (2) $$\begin{array}{l} d\left[k\right]=\frac{\hat{a}\left[k+1\right]-\hat{a}\left[k-1\right]}{2},0<k<K \end{array}$$
  (3) $$\begin{array}{l} d\left[0\right]=\hat{a}\left[1\right]-\hat{a}\left[0\right] \end{array}$$
  (4) $$\begin{array}{l} d\left[K\right]=\hat{a}\left[K\right]-\hat{a}\left[K-1\right] \end{array}$$
3. **Time lapse calculation**
  A threshold on the maximum allowed variation in the derivative of the APD time series for convergence to be attained was defined in this study by setting θ = 0.5 ms. The number of cycles, *k*_APD_, for APD convergence following β-AS and/or stretch was defined as:
  (5) $$\begin{array}{ll} k_{\text{APD}} & =\underset{0\leq k\leq K}{\min}\left\{|\overset{K}{\underset{k^{\prime }=k}{\sum }}d\left[k^{\prime }\right]|<\theta \right\} \end{array}$$
  The time lapse τ_APD_ was obtained by converting *k*_APD_ into minutes:
  (6) $$\begin{array}{l} \tau_{\text{APD}}=k_{\text{APD}}\frac{CL}{60} \end{array}$$
  where *CL* is the cycle length in seconds (constant period between stimuli applied to the cells to elicit APs).
  Values of τ_APD_ equal to 0 represent cases where convergence of the APD time series was immediate.

## 3. Results

### 3.1. Time Lapse for Development of LF Oscillations in APD

**Figure 1:**
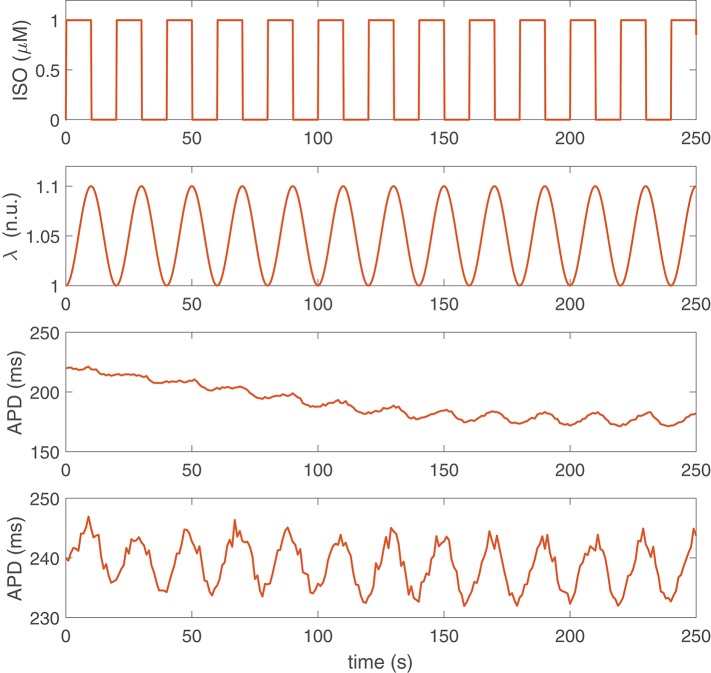
Simulation of sympathetic provocation and APD response of two different cells in the population. First row: Phasic ISO application at a frequency of 0.05 Hz. Second row: Phasic stretch ratio variations at the same frequency. Third and fourth rows: APD time series corresponding to two cells (virtual cell 1 and virtual cell 2) presenting LF oscillations in response to sympathetic provocation.

Figure 1 shows examples of APD time series for two different human ventricular cells of our simulated population presenting LF oscillations following sympathetic provocation. From this figure, it is clear that not only the magnitude of the oscillations is different for the two cells but also the time lapse required for LF oscillations of APD to become evident is remarkably distinct. For the first virtual cell illustrated in Figure 1, the time lapse was τ_APD_ = 139 s, whereas for the second virtual cell, τ_APD_ = 0 s. The characteristics of these two cells in terms of ionic current conductances are presented in Table 2.

**Table 2:** Factors multiplying ionic conductances of virtual cells 1 and 2 illustrated in Figure 1.

**Ionic factors**	**θ**_***Ks***_	**θ**_***Kr***_	**θ**_***to***_	**θ**_***CaL***_	**θ**_***K*1**_	**θ**_***Na***_	**θ**_***NaCa***_	**θ**_***NaK***_
Virtual cell 1	1.83	0.88	0.78	0.46	1.16	1.70	0.40	1.37
Virtual cell 2	0.49	1.11	1.98	1.37	1.34	0.42	1.82	1.97

**Figure 2:**
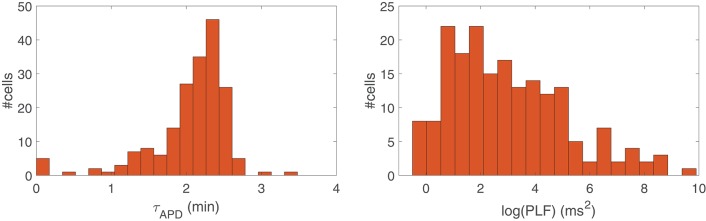
Histogram of the time lapse (**Left**) and LF power (**Right**) of APD in response to increased sympathetic activity for all cells in the simulated population.

Figure 2, left panel, presents a histogram of the time lapse for APD oscillations developed in response to a rise in sympathetic activity for all the cells in our virtual population. Inter-individual differences in the ionic characteristics of the virtual cells had an impact on τ_APD_, which ranged from just a few seconds for some virtual cells to more than 3 min for other cells. Similarly, Figure 2, right panel, shows a histogram of the power in the LF band (PLF) for APD oscillations under sympathetic provocation, represented in terms of log(PLF). Large inter-individual variability also exists in log(PLF), with values covering from 0 to 10 ms^2^, although most cells present PLF values below 5 ms^2^.

### 3.2. Contribution of β-AS and Mechanical Stretch to Time Lapse of LF Oscillations in APD

**Figure 3:**
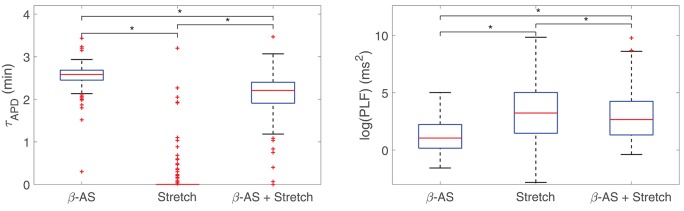
Boxplots representing the time lapse **(left)** and the power in the LF band **(right)** for oscillations of APD to develop in response to phasic β-AS (ISO 1 μM), mechanical stretch (10%) and the combination of both. Statistically significant differences by Wilcoxon signed-rank test (*p*-value < 0.05) are denoted by *. Since the statistical significance in the comparison of simulated data highly depends on the number of simulated cases, smaller subsets of virtual cells were used to prove that *p* < 0.05 had already been achieved with a much smaller number of virtual cells than those in the whole population.

The individual and combined contributions of phasic β-AS and mechanical stretch to the time lapse in the occurrence of LF oscillations of APD is presented in Figure 3, left panel. As can be observed from the figure, individual application of phasic β-AS had a major role in the time required for APD oscillations to develop, whereas individual mechanical stretch had a more marginal influence, with the vast majority of simulated cells developing LF oscillations in response to phasic stretch in less than 1 min. When the effects of β-AS and stretch were combined, the APD convergence time was reduced with respect to that corresponding to only β-AS for practically all cells.

Additionally, Figure 3, right panel, illustrates the oscillation magnitudes in terms of log(PLF) for individual and combined β-AS and mechanical stretch. Individual mechanical stretch led to the largest oscillations magnitudes, in association with the shortest time delays, whereas individual β-adrenergic stimulation led to the smallest magnitudes, in association with the largest time lapses. Nevertheless, high inter-individual variability could be observed in all cases.

### 3.3. Comparison of APD Time Lapse Following β-AS in Experiments and Simulations

Based on the results presented in sections 3.1 and 3.2 and the fact that LF oscillations of APD are superimposed to the general trend of APD decrease following enhanced sympathetic activity, the time lapse for the development of APD oscillations can equivalently be determined by the time required for APD to converge to steady-state following constant β-AS.

**Figure 4:**
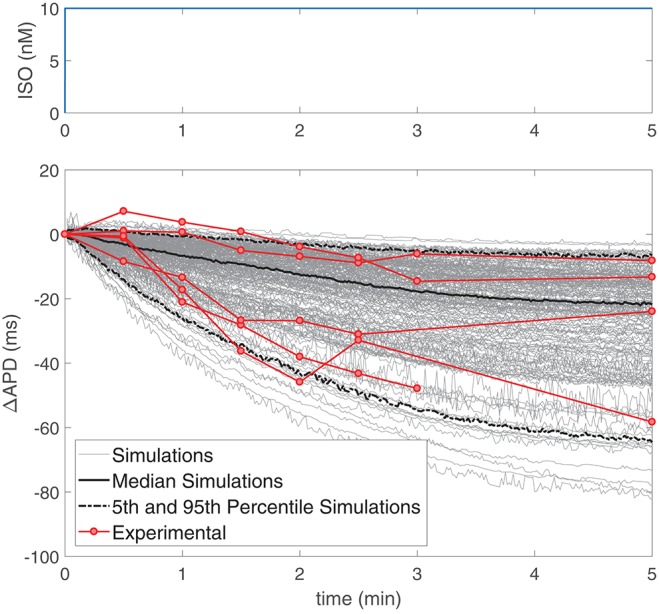
(**Top**) ISO dose in nM, where time zero indicates the time when the solution containing ISO arrived to the cells and analogously for simulations. (**Bottom**) Change in APD with respect to baseline following application of a constant 10 nM ISO dose in experiments (*n* = 5, red) and simulations (gray) on single ventricular myocytes.

The temporal evolution of APD following constant application of an ISO dose of 10 nM was investigated in simulations based on our generated population of cells and compared with our experimental data recorded by using the same β-AS protocol with the same ISO dose. Figure 4 presents ΔAPD, calculated by subtracting the mean APD value at baseline (prior to ISO application) to the APD time series measured following β-AS, for both simulated and experimental data from single ventricular myocytes. It can be noted from the figure that large cell-to-cell variability exists in the time lag of measured APD responses, with the transition times required to reach steady-state following ISO application varying by several minutes. This cell-to-cell heterogeneity in the APD response to constant β-AS serves as a basis to explain the cell-to-cell differences in the data presented in Figure 3 (left column), corresponding to phasic β-AS at a 1 μM ISO dose, which includes APD oscillations overlapped with the decrease in APD. Of note, the simulated time lags in our virtual population of cells are representative of the values measured experimentally in ventricular cardiomyocytes.

### 3.4. Reduction in Time Lapse for LF Oscillations of APD by Prior Low-Level β-AS

**Figure 5:**
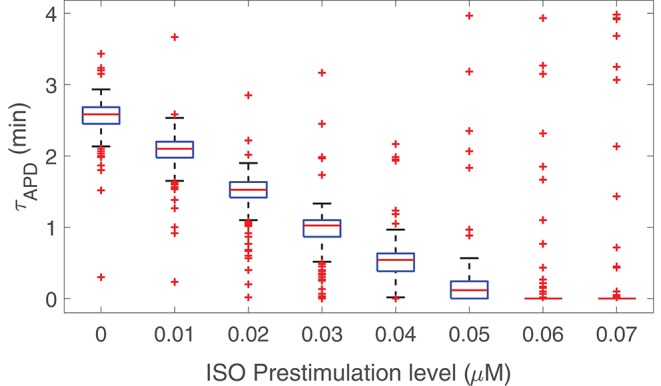
Time lapse for LF oscillations of APD to develop in response to phasic β-AS with 1 μM ISO dose as a function of prior phasic β-AS with lower ISO doses varying from 0 to 0.07 μM.

The possibility that prior stimulation of β-adrenoceptors could reduce the time required for APD to develop LF oscillations in response to enhanced sympathetic activity was next explored. Figure 5 presents results of the time lapse for oscillations development in response to phasic 1 μM ISO application for eight different cases with prior β-AS corresponding to ISO levels varying from 0 to 0.07 μM in 0.01 μM-steps, with each of these pre-stimulation periods applied for 500 beats at 1 Hz pacing frequency. From this figure, it is clear that the time lapse was remarkably reduced as a function of the pre-stimulation level. For a prior stimulation with an ISO dose of 0.05 μM, i.e., 50 nM, most virtual cells developed LF oscillations in APD practically in an instantaneous way after applying the maximal ISO dose of 1 μM. There are still some cells for which the time lapse is above 3 min even if β-adrenoceptors were previously stimulated. Pre-stimulation did not have any remarkable effect on the magnitude of the APD oscillations.

### 3.5. Ionic Mechanisms Underlying Time Lapse in LF Oscillations of APD

**Figure 6:**
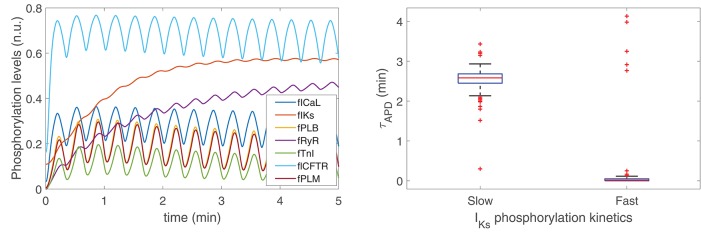
(**Left**) Phosphorylation levels calculated as described in section 2.3. (**Right**) Time lapse for LF oscillations of APD to develop in response to phasic β-AS when using PKA models with slow [**Left**, Xie et al. (2013)] and fast [**Right**, Soltis and Saucerman (2010)] *I*_*Ks*_ phosphorylation and dephosphorylation kinetics.

To ascertain the ionic mechanisms underlying the time required for APD to develop LF oscillations following phasic β-AS, the effect of phosphorylation and dephosphorylation kinetics of all cellular PKA substrates was investigated. Figure 6, left panel, presents the phosphorylation levels of all these substrates in response to 5 min adrenergic stimulation. As can be observed from the figure, the substrates presenting slower phosphorylation responses are the slow delayed rectifier channels, associated with the *I*_*Ks*_ current, and ryanodine receptors, RyR.

To assess the extent to which variations in the phosphorylation and dephosphorylation kinetics of *I*_*Ks*_ influenced the time for development of APD oscillations, simulations were run where the *I*_*Ks*_ phosphorylation and dephosphorylation rate constants were increased to the values described in Soltis and Saucerman (2010) from which an update was presented in a subsequent study by Xie et al. (2013) to more reliably recapitulate PKA-dependent regulation of *I*_*Ks*_. Specifically, the *I*_*Ks*_ phosphorylation rate constant was changed from 8.52 to 84 s^−1^ and the *I*_*Ks*_ dephosphorylation rate constant was changed from 0.19 to 1.87 s^−1^. According to the results presented in Figure 6, right panel, it is clear that the time lapse for APD oscillations was very notably reduced after increasing those rate constants, thus indicating the dependence of the APD oscillatory time lapse on *I*_*Ks*_ phosphorylation kinetics. On the other hand, variations in the phosphorylation kinetics of RyR had no impact on the time lapse for APD oscillations to develop, even if these were varied by a factor of up to ten times their nominal values.

Based on the above results, and considering that cell-to-cell differences in our population of models correspond to different ionic current conductance contributions, it was hypothesized that inter-individual differences in the time lapse for APD oscillation development was based on their differential *I*_*Ks*_ contributions. Simulations were run where *I*_*Ks*_ was inhibited at different levels and a monotonic decrease in oscillation time lapse could be quantified for increasingly larger inhibitions, as illustrated in Figures S1, S2. For full *I*_*Ks*_ blockade, APD oscillations became apparent almost immediately.

### 3.6. Relationship Between Time Lapse and Magnitude of LF Oscillations of APD

**Figure 7:**
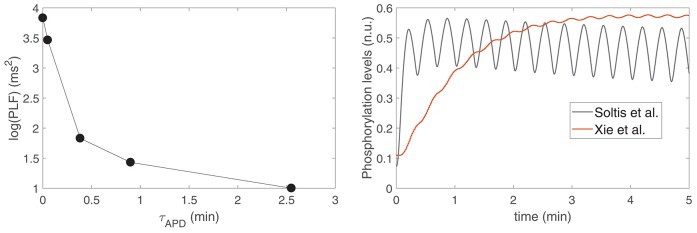
(**Left**) PLF vs. τ_APD_ for varying *I*_*Ks*_ phosphorylation and dephosphorylation rate constants ranging from the values in Soltis and Saucerman (2010) to the values in Xie et al. (2013). (**Right**) *I*_*Ks*_ phosphorylation levels for the models with *I*_*Ks*_ phosphorylation and dephosphorylation rate constants as in Soltis and Saucerman (2010) (gray line) and as in Xie et al. (2013) (red line).

To assess the relationship between the time lapse for development of LF oscillations in APD and the magnitude of such oscillations, a set of models was built in such a way that they all share the same characteristics of the ORd-Xie coupled electrophysiology-β-adrenergic signaling model, except for *I*_*Ks*_ phosphorylation and dephosphorylation rate constants, which were varied from model to model so that they covered from the slowest dynamics reported in Xie et al. (2013) to the fastest dynamics reported in Soltis and Saucerman (2010). Figure 7, left panel, shows the relation between the magnitude of LF oscillations in APD, quantified by the LF power in the 0.04–0.15 Hz band denoted by PLF, and the time lapse for oscillation development, quantified by τ_APD_. It can be observed from the figure that the models with the fastest *I*_*Ks*_ phosphorylation dynamics are those presenting the shortest time lapse and the highest APD oscillatory magnitude.

To substantiate this result, Figure 7, right panel, shows *I*_*Ks*_ phosphorylation levels calculated according to the signaling models in Xie et al. (2013) and Soltis and Saucerman (2010), corresponding to the two most extreme points shown in Figure 7, left panel. It can be observed from the graphic that, for the model in Soltis and Saucerman (2010), not only are the *I*_*Ks*_ phosphorylation dynamics faster but also the associated oscillations are of larger magnitude. These enhanced oscillations in *I*_*Ks*_ phosphorylation have an impact on the AP, which is manifested by a larger oscillatory magnitude of APD.

In the whole population of virtual cells, where all cells present the same phosphorylation kinetics but the conductance of *I*_*Ks*_ varies from one cell to another, consequently modulating the influence of *I*_*Ks*_ phosphorylation fluctuations on APD oscillatory behavior, the inverse relationship between PLF and τ_APD_ can still be appreciated. This is shown in **Figure 10**, which presents PLF vs τ_APD_ for cells under healthy conditions divided into two groups depending on the presence/absence of pro-arrhythmic effects when disease conditions were simulated, as described in the next section.

### 3.7. Effect of Disease Conditions in Time Lapse of LF Oscillations of APD and Relation to Arrhythmogenesis

**Figure 8:**
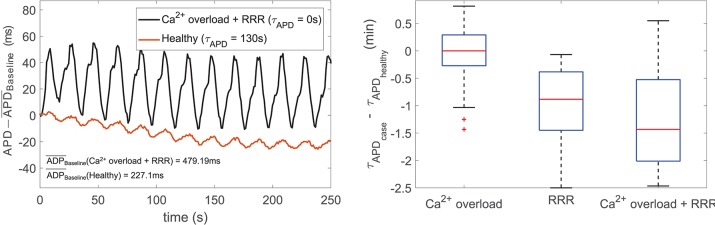
(**Left**) Zero-mean APD series (APD - ${\bar{\text{APD}}}_{\text{Baseline}}$) in response to sympathetic provocation, for healthy (red line) and disease (black line) conditions simulated for a virtual cell of the population. (**Right**) Differences in τ_APD_ due to Ca^2+^ overload and/or RRR with respect to healthy conditions.

Simulation of disease conditions by Ca^2+^ overload and RRR in our population of models led to a sharp decrease in the APD oscillatory time lapse following increased sympathetic activity. This is illustrated in Figure 8, left panel, which shows zero-mean APD time series (after subtraction of the corresponding baseline value to facilitate comparison) for one of the cells in the virtual population under healthy and pathological conditions. The value of τ_APD_ decreased from 130 to 0 ms due to the effects of disease. Figure 8, right panel, summarizes the observed changes in τ_APD_ when simulating disease conditions in the subpopulation of cells that did not present pro-arrhythmic events. Whereas Ca^2+^ overload had mild effects on τ_APD_, the effects of RRR, individually or in the presence of Ca^2+^ overload, contributed to a very remarkable reduction in the oscillatory time lapse.

**Figure 9:**
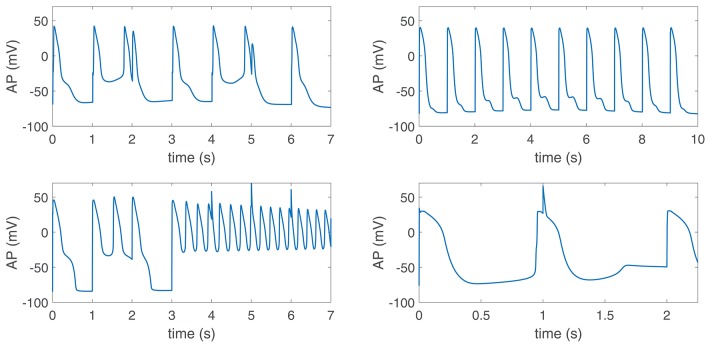
Pro-arrhythmic events in virtual cells in response to increased sympathetic activity under diseased conditions simulated by Ca^2+^ overload, reduced repolarization reserve and increased G_*SAC*_. Phase 2 and phase 3 early afterdepolarizations (EADs) **(top panels)**, EAD bursts **(bottom left)** and spontaneous beats **(bottom right)** could be observed.

**Figure 10:**
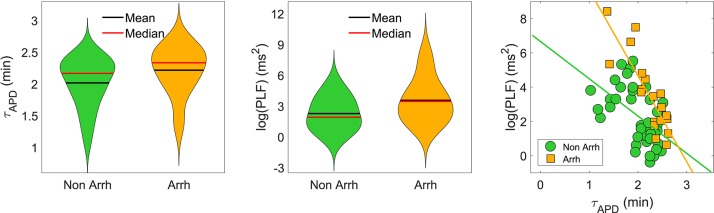
**(Left and middle)**: Violin representations of τ_APD_ and log(PLF), respectively, calculated under healthy conditions for subpopulations of cells presenting and not presenting pro-arrhythmic events when disease conditions were simulated while pacing at CLs of 1,000, 2,000, and 2,500 ms. **(Right)** τ_APD_ vs. log(PLF) for the same two subpopulations. The slopes of the regression lines for the subpopulations presenting (orange) and not presenting (green) pro-arrhythmic events were statistically significantly different by univariate analysis of variance (*p*-value < 0.05).

When disease conditions were simulated as accompanied by an increase in the conductance of non-specific cationic SACs in accordance with experimental evidences (Kamkin et al., 2000; Guinamard et al., 2006), arrhythmogenic events were generated in some of the virtual cells of the population following sympathetic provocation. These were in the form of afterdepolarizations and spontaneous beats and occurred in 46.34% of the virtual cells that did not show any pro-arrhythmic manifestation at baseline. Examples are illustrated in Figure 9. To assess whether individual cell oscillatory characteristics evaluated under healthy conditions were related to pro-arrhythmicity, the time lapse, quantified by τ_APD_, and the magnitude of APD oscillations, quantified by PLF, were compared between the groups of cells presenting and not presenting arrhythmogenic events. Results are presented in Figure 10, left and middle panels. As can be observed from the figure, little differences in the mean or median τ_APD_ were found between the two groups. On the other hand, larger differences in PLF were seen between the groups, with the one presenting arrhythmogenic events in response to increased sympathetic activity being associated with remarkably larger mean and median PLF (note that the logarithm of PLF is represented in Figure 10). Boxplots of τ_*APD*_ and log(PLF) for the groups of cells presenting and not presenting arrhythmogenic events are shown in Figure S3.

The relationship between PLF and τ_APD_ in the population of cells prior to introducing disease conditions is presented in Figure 10, right panel, for the pro-arrhythmic and non-pro-arrhythmic groups. In both groups, larger values of PLF were associated with shorter values of τ_APD_, although high inter-individual variability could be noticed. The Spearman correlation coefficient was ρ = −0.82 in the pro-arrhythmic group and ρ = −0.57 in the non-pro-arrhythmic group.

## 4. Discussion

### 4.1. Inter-individual Differences in the Time Lapse for Development of LF Oscillations of APD Following Enhanced Sympathetic Activity

The research presented in this study has shown that LF oscillations of human ventricular repolarization, reported in the T-wave of the ECG and locally in ARIs of unipolar epicardial electrograms, do not develop immediately upon a sympathetic rise but take some time to become apparent. An algorithm has been proposed to robustly quantify the time lapse required for APD to develop sympathetically-mediated LF oscillations. This time lapse has been shown to be highly variable from one cell to another, ranging from just a few seconds to more than 3 min depending on the ionic characteristics of each individual cell. Following enhanced sympathetic activity, the APD shows a trend of shortening, or brief prolongation followed by more sustained shortening, which masks overlapping oscillations. Only when such APD shortening has been completed, APD oscillations become manifest.

The range of time lags for APD oscillatory behavior following sympathetic provocation is of the order of adaptation lags reported for the QT interval of the ECG in response to increases in sympathetic activity leading to abrupt heart rate increases, either measured from ambulatory Holter recordings (Pueyo et al., 2004) or following tilt test (Pueyo et al., 2008; Nosakhare et al., 2014). Those repolarization dynamics have also been recently investigated in experimental studies using fully innervated Langendorff-perfused mouse and rabbit hearts, where the APD response to bilateral sympathetic nerve stimulation has been described (Wang et al., 2019). In those studies ventricular repolarization was modulated both by direct sympathetic action on the ventricular myocardium as well as indirectly by heart rate-related effects. In the present study, CL was kept constant and the ventricular response was thus only assessed as due to sympathetic effects on the ventricle, as in *in vivo* electrogram recordings from patients where LF oscillations of ARI have been characterized while controlling CL with right ventricular pacing (Hanson et al., 2014; Porter et al., 2018).

The prolonged time lapses for LF oscillatory behavior of APD following enhanced sympathetic activity quantified in this study can help to explain why oscillations seem to appear and disappear, as observed in *in vivo* studies (Hanson et al., 2014), where APD oscillatory behavior could only be measured at certain time intervals of the analyzed recordings. Those time intervals could be speculated to be associated with sustained sympathetic activation so that enough time was allowed for LF oscillations in APD to develop.

In this work sympathetic provocation was simulated by concomitant phasic changes in β-AS and mechanical stretch. The involvement of each of these two components in the protracted LF oscillatory response to a sympathetic rise has been assessed. Our results have determined that mechanical stretch induces LF oscillations of APD in an almost instantaneous manner, whereas β-AS entails much longer APD time courses until LF oscillations can be clearly appreciated. Based on the fact that the time lapse is mainly due to the slow response to β-AS, this study has next validated the calculated time lapses against *in vitro* data from ventricular myocytes following sudden exposure to ISO. Both in the experiments and the simulations of this study, the time required for APD to reach steady-state following sudden β-AS was found to highly vary from cell to cell. Simulated time lapses were comprised within the experimental limits quantified for the ventricular myocytes of this and other studies (Liu et al., 2012; Ruzsnavszky et al., 2014), thus confirming validation of our population of models to reproduce available evidences on the APD time course in response to β-AS.

To further support our conclusions on the key role of β-AS in determining the time lapse for LF oscillations of APD to develop, the effects of pre-stimulating ventricular cells with a lower dose of the β-adrenergic agonist ISO have been tested. Results have confirmed that the oscillatory time lapse is highly dependent on β-adrenoceptors' state. The higher the prior stimulation level of β-adrenoceptors, the shorter the time for development of LF oscillations. This reduction in the oscillatory time lapse by prior ISO exposure agrees with common knowledge on pre-stimulation of β-adrenoceptors altering the impact of β-AS. Under conditions associated with high sympathetic tone, as in failing or aged ventricles, sympathetic surge would thus be expected to induce LF oscillations of repolarization with shorter latency. Consequently, due to the less stringent requirements on the time period of sustained sympathetic activation for LF oscillatory behavior to ensue in failing or aged ventricles, this is anticipated to facilitate the occurrence of such oscillations, with the corresponding potentially adverse consequences (Rizas et al., 2014, 2017; Pueyo et al., 2016; Sampedro-Puente et al., 2019).

### 4.2. Major Role of *I*_*Ks*_ Phospohorylation Kinetics in Determining the Time Lapse for LF Oscillations of APD

The mechanisms underlying the slow appearance of APD oscillations following sympathetic provocation, particularly related to the protracted response to β-AS, have been ascertained in this work by comparing the phosphorylated levels of all cellular substrates accounted for in the modified β-adrenergic signaling model by Xie et al. (2013) used as a basis for this study. Two cellular substrates, namely *I*_*Ks*_ and RyR, have been shown to present responses to β-AS being remarkably slower than those of all other substrates. The time required for *I*_*Ks*_ and RyR phosphorylation levels to reach steady-state upon β-AS is around 3 min, this being close to the maximum time lapse for APD oscillations to appear in our simulated population of models, while the phosphorylation levels of the remaining cellular substrates reach steady-state in no more than 20–30 s. In other β-adrenergic signaling models, as in the model by Heijman et al. (2011), *I*_*Ks*_ and RyR present slow kinetics too, although there are other substrates, like the Na^+^-K^+^-ATPase current, with even slower kinetics.

The impact of the slow *I*_*Ks*_ and RyR phosphorylation kinetics on the APD time course following sympathetic stimulation has been assessed by varying their phosphorylation and dephosphorylation rate constants. Whereas variations in the kinetics of *I*_*Ks*_ are proved to have relevant effects on the time lapse for APD oscillations, the influence of variations in the RyR kinetics is negligible. The irrelevant role of RyR phosphorylation on τ_APD_ as compared to that of *I*_*Ks*_ phosphorylation can be explained on the basis of their very distinct impact on APD. RyR phosphorylation has been described in this study according to the formulation proposed in Heijman et al. (2011), where it has been shown that disabling RyR phosphorylation leads to little variations in APD with respect to measurements when all substrates are phosphorylated. On the other hand, *I*_*Ks*_ phosphorylation has much more prominent effects on APD (Xie et al., 2013). To further support the role of *I*_*Ks*_ in determining the APD oscillatory latency, this current has been inhibited to various extents and it has been confirmed that the larger the *I*_*Ks*_ current amplitude, the longer the latency. These results lead us to conclude that the high inter-individual variability in the time lapse for APD oscillations characterized in our population of models can be explained by differential *I*_*Ks*_ contributions from one cell to another.

The important role of *I*_*Ks*_ during β-AS has been pointed out in numerous studies (Volders et al., 2003; Johnson et al., 2010, 2013; Hegyi et al., 2018; Varshneya et al., 2018). Reduced *I*_*Ks*_ responsiveness to β-AS has been suggested to increase arrhythmia susceptibility in a heart failure animal model (Hegyi et al., 2018). In ventricular myocytes, loss of *I*_*Ks*_ current has been experimentally shown to exaggerate beat-to-beat APD variability in response to β-AS (Johnson et al., 2010, 2013) and computationally proved to facilitate the generation of pro-arrhythmic early afterdepolarizations (Varshneya et al., 2018). Our results provide additional support to the role of *I*_*Ks*_ during β-AS, as reduced *I*_*Ks*_ shortens the oscillatory latency and thus facilitates the occurrence of LF oscillations of APD. This oscillatory behavior of ventricular repolarization can be seen as a particular form of beat-to-beat variability restricted to frequencies in the Mayer wave frequency range (0.03–15 Hz).

### 4.3. Increased Arrhythmic Risk as a Function of the Time Lapse and Magnitude of LF Oscillations of APD

RRR, individually or combined with Ca^2+^ overload, has been found to dramatically reduce the time lapse for sympathetically-induced oscillatory behavior. This can be understood on the basis that under RRR the amount of *I*_*Ks*_ current is reduced and, provided phosphorylation kinetics are not varied, this leads to a reduction in the oscillation time lag of the APD. Since the above holds for each of the virtual cells in the population built this study, the time lapse values measured under pathological conditions are lower than the ones corresponding to non-pathological conditions.

A comparison for time lapses calculated for cells under healthy conditions has been established while considering two groups of interest, one composed of cells presenting and the other one not presenting arrhythmogenic events after simulation of disease conditions. Results have been shown to be comparable. However, in both the pro-arrhythmic and non-pro-arrhythmic groups, there is an inverse relationship between the magnitude of LF oscillations of APD, measured by PLF, and the time required for such oscillations to develop. These findings indicate that cells in which APD oscillations appear rapidly in response to enhanced sympathetic activity are associated with larger oscillatory magnitudes. Although the inverse relationship between PLF and the oscillatory time lapse holds true for both groups, such a relationship is steeper in the pro-arrhythmic group, with given low time lapse values associated with larger oscillatory magnitudes. Those enhanced magnitudes may facilitate the occurrence of arrhythmic events that can act as triggers for arrhythmias and at the same time they may contribute to a more vulnerable substrate by increasing spatial repolarization inhomogeneities between regions being at different oscillating phases. This increased arrhythmia susceptibility associated with elevated LF oscillations of repolarization has been postulated by *in silico* studies (Pueyo et al., 2016; Sampedro-Puente et al., 2019) and confirmed by *in vivo* research on a CAVB dog model (Sprenkeler et al., 2019) as well as clinical studies in post-infarction patients. (Rizas et al., 2017). These results are in line with studies associating higher levels of temporal repolarization variability, in the form of alternans or in other forms, with increased arrhythmic risk (Rosenbaum, 2001; Porter et al., 2019).

The role of *I*_*Ks*_ expression and phosphorylation dynamics in pro-arrhythmia that has been uncovered in the present study is in line with previous studies investigating ventricular repolarization response to β-AS. The slow *I*_*Ks*_ phosphorylation kinetics as compared to the fast *I*_*Ca*_ kinetics have been reported to be behind the generation of transient arrhythmogenic early afterdepolarizations (Liu et al., 2012; Xie et al., 2013) and APD alternans (Xie et al., 2014b) upon sudden ISO application. In our study, the fact of simulating a whole population of cells allows to additionally reveal the importance of *I*_*Ks*_ conductance in determining τ_APD_, as *I*_*Ks*_ conductance modulates the relevance of *I*_*Ks*_ dynamics on APD time course during β-AS. Additionally, differential *I*_*Ks*_ and *I*_*Ca*_ activation kinetics in response to sudden β-AS have been shown to promote the transition from ventricular tachycardia to ventricular fibrillation by transiently steepening APD restitution in simulated ventricular tissues (Xie et al., 2014a). This same ionic mismatch has been suggested as a plausible mechanism underlying a transitory increase in the risk for arrhythmias by application of sudden adrenergic stress in isolated innervated rabbit hearts treated with a potassium channel blocker and subjected to sustained parasympathetic stimulation (Winter et al., 2018).

### 4.4. Study Limitations

In this study, simulations have been run to quantify the time lapse for development of sympathetically-mediated LF oscillations of APD in a large population of human ventricular AP models developed based on available experimental data. After confirming the role of β-AS, over the role of mechanical stretch, in determining such oscillatory time lapse, our simulated results were compared with available *in vitro* data from isolated canine ventricular myocytes in response to sudden administration of a β-adrenergic agonist. Despite differences between species, experimental studies have shown that ventricular repolarization characteristics of canine cardiomyocytes closely resemble those of human cardiomyocytes (Szabó et al., 2005; Szentandrássy et al., 2005). If additional *in vitro* and/or *in vivo* data became available to analyze the time required for ARI or APD oscillations to become manifest following sympathetic provocation, further validation of the results obtained in the present study could be performed.

The simulated results presented in this study correspond to single cells. As a continuation of this investigation, tissue models built on the basis of the present population of AP models could be used to assess whether other tissue-specific factors could play a relevant role in the time required for APD oscillations to develop, in the magnitude of such oscillations as well as in the associated consequences in terms of pro-arrhythmic risk.

The population of human ventricular computational models built in this study used the O'Hara et al. (2011) model as a basis to describe human ventricular electrophysiology and calcium dynamics, whereas mechanics were described by a modified version of the Niederer et al. (2006) model. For β-adrenergic signaling, the Xie et al. (2013) model was used as a basis and the Soltis and Saucerman (2010) model was used for additional comparisons. These selections might have an impact on the conclusions reached in this study, particularly regarding quantitative values for the time required for LF oscillations of APD to develop. Nevertheless, in Pueyo et al. (2016), different human and animal cell models were tested for APD oscillatory behavior, confirming model-independence in qualitative terms with only some quantitative differences between different electrophysiological models, particularly for different species. Future studies could address the investigations conducted in this study while using other cellular models as a basis for construction of a population of models representative of human or animal ventricular electrophysiological characteristics reported experimentally and compare with the results of this study.

The developed population of human ventricular AP models was deterministic. Future work could include incorporation of stochasticity into the main ionic currents active during AP repolarization. This would allow accounting for beat-to-beat repolarization variability, which might have an effect in the time course for development of LF oscillations of APD.

An ISO dose of 0 μM was used to represent β-AS under baseline conditions. Although results are anticipated to be very similar to those obtained for a low ISO dose slightly above 0, somewhat different time lapse values for APD oscillations might be quantified.

## 5. Conclusions

Human ventricular repolarization presents low-frequency oscillations that develop following enhanced sympathetic activity at time lapses varying from a few seconds to more than 3 min depending on individual cells characteristics. The latency in the oscillatory development is due to the slow ventricular response to β-adrenergic stimulation and, specifically, it is associated with the slow phosphorylation kinetics of the *I*_*Ks*_ current. Prior stimulation of β-adrenoceptors reduces the time required for the development of repolarization oscillations. Short time lapses are associated with large APD oscillatory magnitudes, particularly in cells susceptible to develop arrhythmogenic events in response to sympathetic stimulation.

## Data Availability Statement

The datasets generated for this study are available on request to the corresponding author.

## Author Contributions

EP and PT devised the project, the main conceptual ideas and proof outline, and were responsible for overseeing the research and providing critical insight and recommendations regarding the focus, structure and content of the paper. DS-P and JF-B performed computational simulations and analyzed the data results. NS and PN contributed with technical details and analysis support. All authors participated in writing and proofreading throughout the publication process.

### Conflict of Interest

The authors declare that the research was conducted in the absence of any commercial or financial relationships that could be construed as a potential conflict of interest.
