# Supplementary material for: Time Course of Low-Frequency Oscillatory Behavior in Human Ventricular Repolarization Following Enhanced Sympathetic Activity and Relation to Arrhythmogenesis
Source: Front Physiol. 2020 Jan 14;10:1547. doi: 10.3389/fphys.2019.01547 (PMC6971219; doi:10.3389/fphys.2019.01547)
Supplement: Supplementary file 1 [file Data_Sheet_1.PDF]

Supplementary Material:

Time Course of Low-Frequency Oscillatory Behavior in Human Ventricular Repolarization Following Enhanced Sympathetic Activity and Relation to Arrhythmogenesis

1 SUPPLEMENTARY TABLES AND FIGURES

1.1 Figures

1.1.1 Simulation of I_{Ks} block under individually β-AS and in combination with Mechanical Stretch

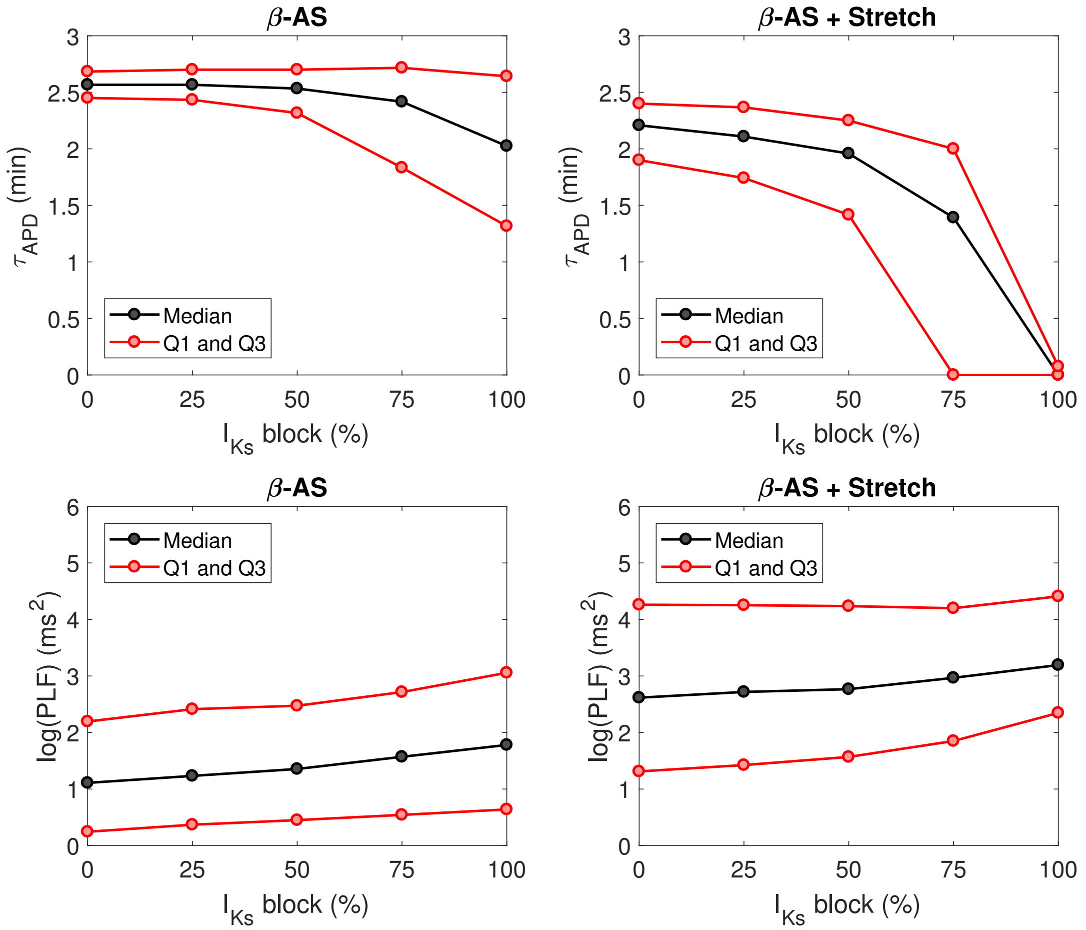

Figure S1. τ_{APD} (top panels) and log(PLF) (bottom panels), presented in terms of median, first quartile (Q1) and third quartile (Q3), for increasingly higher degrees of I_{Ks} inhibition, both in response to phasic β-AS (ISO 1μM, left panels) and combined with phasic mechanical stretch (10%, right panels) for the population of virtual cells under healthy conditions.

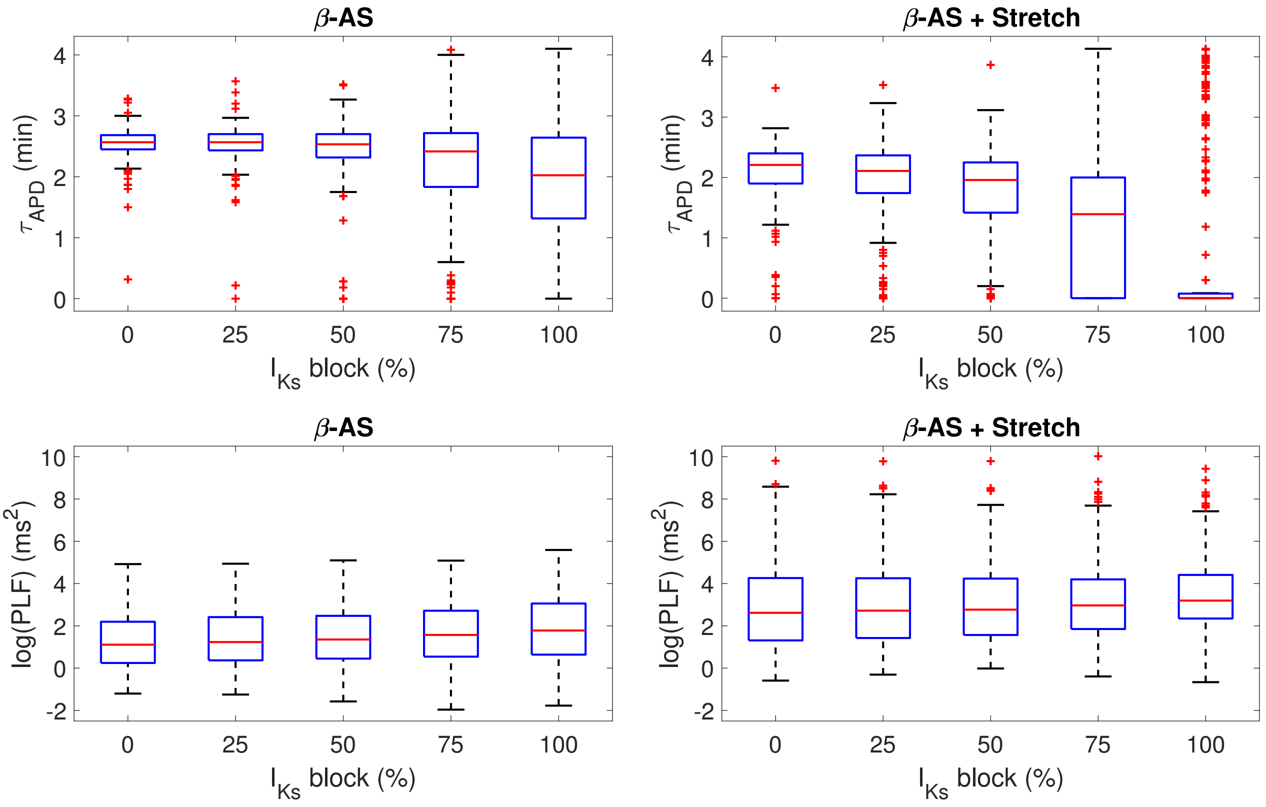

Figure S2. Boxplots of τ_{APD} (top panels) and log(PLF) (bottom panels) for increasingly higher levels of I_{Ks} inhibition, both in response to phasic β-AS (ISO 1μM, left panels) and combined with phasic mechanical stretch (10%, right panels) for the population of virtual cells under healthy conditions.

1.1.2 Effect of disease conditions in time lapse of LF oscillations of APD and its relationship with arrhythmogenesis

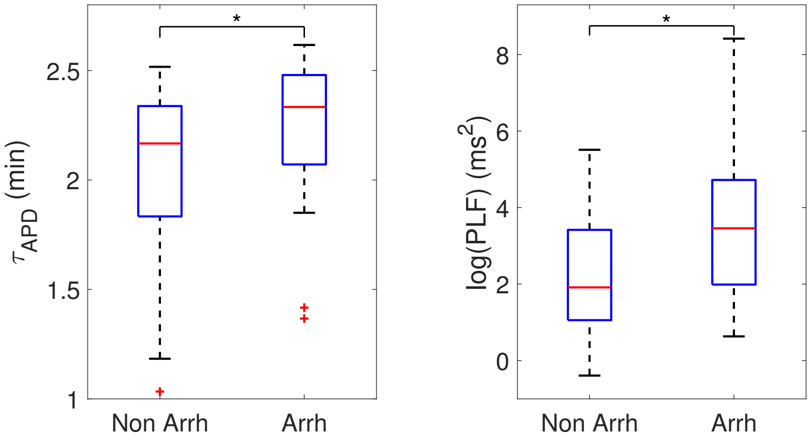

Figure S3. Boxplot of τ_{APD} (left panel) and log(PLF) (right panel) calculated under healthy conditions for subpopulations of cells presenting and not presenting pro-arrhythmic events when disease conditions were simulated while pacing at CLs of 1000, 2000 and 2500 ms. Statistically significant differences by Wilcoxon rank-sum test (p-value < 0.05) are denoted by $*$, while non-significant differences are denoted by $n.s$. See comment on statistical comparisons of simulated data in the main manuscript.
